# Development of Rifampicin-Indocyanine Green-Loaded Perfluorocarbon Nanodroplets for Photo-Chemo-Probiotic Antimicrobial Therapy

**DOI:** 10.3389/fphar.2018.01254

**Published:** 2018-11-02

**Authors:** Kuang-Hung Hsiao, Chun-Ming Huang, Yu-Hsiang Lee

**Affiliations:** ^1^Department of Biomedical Sciences and Engineering, National Central University, Taoyuan City, Taiwan; ^2^Department of Dermatology, University of California, San Diego, San Diego, CA, United States; ^3^Department of Chemical and Materials Engineering, National Central University, Taoyuan City, Taiwan

**Keywords:** *P. acnes*, indocyanine green, perfluorocarbon, double emulsion, probiotics, photochemoprobiotic therapy

## Abstract

Acne vulgaris, generally resulted from overgrowth of *Propionibacterium acnes* (*P. acnes*), is one of the most difficult-to-treat facial dermatoses and more than 90% of adolescents experienced the disease worldwide. Because the innate non-lymphoid immune system cannot effectively eliminate excessive *P. acnes* from the skin surface, so far the therapy of acne vulgaris is still mainly dependent on antibiotic treatment. However, long-term or overdose of antibiotics may initiate microbial drug resistance and/or generate unexpected side effects that seriously hamper the use of antibiotics in the clinic. To overcome the aforementioned challenges, the novel rifampicin (RIF)-indocyanine green (ICG)-loaded perfluorocarbon (PFC) nanodroplets (RIPNDs) that may offer combined photo-, chemo-, and probiotic efficacies to *P. acnes* eradication were developed in this study. The RIPND was first characterized as a sphere-like nanoparticle with surface charge of −20.9 ± 2.40 mV and size of 240.7 ± 6.73 nm, in which the encapsulation efficiencies of RIF and ICG were 54.0 ± 10.5% and 95.0 ± 4.84%, respectively. In comparison to the freely dissolved ICG, the RIPNDs conferred an enhanced thermal stability to the entrapped ICG, and were able to provide a comparable hyperthermia effect and markedly increased production of singlet oxygen under near infrared (NIR; 808 nm, 6 W/cm^2^) exposure. Furthermore, the RIPNDs were able to induce fermentation of *S. epidermidis* but not *P. acnes*, indicating that the RIPNDs may serve as a selective fermentation initiator for the target probiotics. Based on the microbial population index analyses, *P. acnes* with 1 × 10^6^ cells/mL can be completely eradicated by 12-h co-culture with *S. epidermidis* fermentation products followed by treatment of RIPNDs (≥20-μM ICG/3.8-μM RIF) + NIR for 5 min, whereby the resulted microbial mortality was even higher than that caused by using 16-fold enhanced amount of loaded RIF alone. Overall these efforts show that the RIPNDs were able to provide improved ICG stability, selective fermentability to *S. epidermidis*, and enhanced antimicrobial efficacy compared to equal dosage of free RIF and/or ICG, indicating that the developed nanodroplets are highly potential for use in the clinical anti-*P. acne* treatment with reduced chemotoxicity.

## Introduction

Clinically, acne vulgaris, or commonly known as pimples, remains one of the most difficult-to-treat facial dermatoses, and is often caused by overgrowth of *Propionibacterium acnes* (*P. acnes*) and/or accumulation of excessive inflammatory substances in the hair follicles. According to global disease statistics, acne vulgaris is the 8th most common disease in the world that >600 million people worldwide suffer from the disease every year and more than 90% of adolescents have experienced the dermatosis (GBD 2015 Disease and Injury Incidence and Prevalence Collaborators, 2016). In general, acne vulgaris can be classified in two different types based on the inflammation or not. The one without inflammation is simply caused by blockage of hair follicle (i.e., comedones) which may not damage the basement membrane, whereas the inflammatory type commonly known as papules and/or pustules may hurt the hair follicles or even dermal tissues and form difficult-to-treat nodules and/or cysts afterward if the injury is deteriorated (Kraft and Freiman, 2011).

In terms of the acne therapeutics, it has been demonstrated that the innate non-lymphoid immune system cannot effectively remove excessive *P. acnes* from the skin surface because *P. acnes* are able to survive and grow in deep hypoxic tissues for ≥6 months where the amount of macrophage is relatively low (Csukás et al., 2004). Furthermore, *P. acnes* may resist the phagocytosis (Montes and Wilborn, 1970) or even can survive in the macrophage phagosomes through the protection by its fibrillar layer structure of cell wall (Webster et al., 1985). Therefore, so far the therapy of acne vulgaris is still mainly dependent on the treatment of antibiotics such as clindamycin, erythromycin, rifamycin, and/or the combinations above. However, long-term or over usage of antibiotics may induce microbial drug resistance and/or generate unexpected side effects that highly restrict the applicability of antibiotics in the clinic. Those circumstances indicate that an effective strategy for *acne* therapy (i.e., anti-*P. acnes*) is still urgently needed in nowadays.

Rifampicin (RIF) is one of the US FDA-approved bactericidal antibiotics and its antimicrobial effect is mainly accomplished by inhibition of microbial RNA synthesis (Campbell et al., 2001). Although RIF has been widely used in number of bacteria-borne diseases, the dose of RIF utilized in the clinic is still highly concerned due to its detrimental side effects such as serious redness/itching/pigmentation (Thangaraju et al., 2015) and/or hepatotoxicity (Kunimoto et al., 2003; Garcia-Contreras et al., 2006). Furthermore, repeated oral administration of RIF in high dosage may induce RIF own metabolism and thereby leads to a reduction for its bioavailability (Hiremath and Saha, 2004). To circumvent these issues, co-administration of antimicrobial agents and/or tools is frequently considered as a potential regimen because it may help to decrease the effective dosage of each drug and reduce the potential chemotoxicity accordingly. Among various antibacterial strategies, near infrared (NIR)-based phototherapy has long been identified as a feasible adjuvant in skin medicinal treatment because it may provide (1) less toxicity to normal cells/tissues through use of targeted photosensitive agents and/or spatially controlled light irradiating operation, (2) enhanced tissue penetration effectiveness compared with that performed with UV/visible light, and (3) increased tissue permeability for drug delivery (Cheng et al., 2014; Henderson and Morries, 2015). In general, antimicrobial phototherapy is functionalized by reactive oxygen species (ROS) and/or hyperthermia effect generated from the photosensitizers under light illumination. The ROS is able to damage the microbial membrane and/or metabolism and thus causes cell growth inhibition (photoinactivation) or even death consequently (i.e., photodynamic therapy; PDT), whereas high temperature may cause thermal ablation of microorganism known as photothermal therapy (PTT) (Elman and Lebzelter, 2004). No matter which approach is preferred to utilize, the photosensitizer plays the key role in the effect of phototherapy.

Indocyanine green (ICG), a type of water-soluble tricarbocyanine dye, is the only clinically approved NIR fluorophore with methylene blue. In addition to serving as a contrast agent in many diagnostic applications such as optical coherence tomography-angiography (Mastropasqua et al., 2015) and fluorescence-guided oncologic surgery (Schaafsma et al., 2011), so far ICG is also been widely used for neoplastic and dermatosis phototherapy (Genina et al., 2004; Mundra et al., 2015; Shemesh et al., 2015) due to its capability to generate both heat and singlet oxygen upon NIR exposure. However, quite a few drawbacks of ICG such as rapid plasma clearance (Desmettre et al., 2000) and high aqueous degradability (Saxena et al., 2003) severely hinder its usability in the clinic.

In addition to phototherapy, the methodology of using skin probiotics to affect the growth of to-be-excluded skin microbes has gained increasing attention in the last decade (Iwase et al., 2010; Shu et al., 2013). Such method concept is similar with yogurt-mediated gastrointestinal healthcare that using the probiotics supplied from the yogurt to maintain the ecological balance of the microbes and/or suppress growth of pathogenic microorganism in the intestine (McLoughlin et al., 2017). Shu et al. (2013) have demonstrated that the fermentation products of *P. acnes* was able to effectively inhibit the growth of Methicillin-Resistant *Staphylococcus aureus*. More recently, Wang and his colleagues further showed that the glycerol may selectively accelerate the fermentation of *Staphylococcus epidermidis* (*S. epidermidis*) and the yielded microbial short-chain fatty acids, were able to inhibit the growth of *P. acnes* (Wang et al., 2014). These efforts clearly demonstrate that the probiotics may provide a feasible means for *P. acnes* inhibition. However, the ecology of the commensal microbes on the disease site (i.e., the acne location) should be controlled because any disruption of microbial balance due to over-fermentation of a bacterium (i.e., the one functioned as the probiotics) in a short term may lead to another unpredictable issue.

Nanotechnology/nanomaterial may offer a feasible means for simultaneous use of multi-agents such as RIF and ICG, as well as concurrently provide effect of probiotics-mediated microbial suppression without aforementioned disadvantages because it may provide (1) improved stability and bioavailability to the payloads and (2) controllable fermentation capacity to the selective probiotics based on the dosage used. In this study, we aim to develop a type of RIF-ICG-encapsulated water-in-perfluorocarbon (PFC)-in-water double nanoemulsions; named RIF-ICG-loaded PFC nanodroplets (RIPNDs) to explore the potential of a joint photo-, chemo-, and probiotic therapeutics on acne treatment (i.e., anti-*P. acnes*). PFC, a fluorine-substituted derivative of hydrocarbons, is a well-known robust oxygen transporter since it can dissolve much more respiratory gasses (O_2_ and CO_2_) compared with water (Lowe, 2002). Such feature implies that the PFC constituent will be greatly advantageous for RIPNDs in terms of PDT use. We anticipate that the developed RIPNDs are able to (1) effectively protect the encapsulated ICG from the aqueous degradation caused by external stimuli such as pH, light, and/or heat (Björnsson et al., 1983), (2) selectively enhance fermentation efficiency of *S. epidermidis* but not *P. acnes* to induce probiotics-mediated *P. acnes* inhibition, and (3) provide an effective *P. acnes* eradication with reduced chemotoxicity since the multiplex photo-chemo-probiotic treatment may reduce the effective dosage of the antibiotics performed in the chemotherapy alone. In this paper, we first introduced the fabrication process of the RIPNDs followed by investigating their characteristics, functionalities, and antimicrobial efficacy stepwise.

## Materials and Methods

### Preparation and Characterization of RIPNDs

**FIGURE 1:**
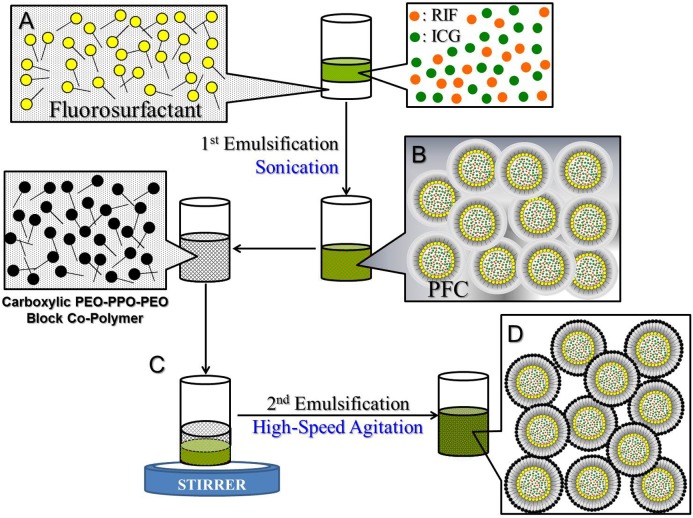
Schematic diagram of the manufacture procedures of the RIPNDs. The RIPNDs structured with fluorosurfactant and carboxylic PEO-PPO-PEO block copolymer were established through a primary sonication (80 W; 10 min; **A** → **B**) followed by a high-speed agitation for an hour (**C** → **D**). To remove excess/unreacted chemicals, the yielded RIPNDs were washed twice with deionized water and stored at 4°C in the dark until use.

The RIPNDs were fabricated using a modified emulsification approach. Briefly, a 500-μL methanol containing RIF [0.04% (w/v)] and ICG [0.1% (w/v)] was first added to a 1-mL perfluorooctyl bromide (PFOB) with 2% (w/w) polyethoxylated fluorosurfactant. The mixture was then subjected to sonication with 80 W in an ice bath for 10 min to obtain the primary water-in-PFC emulsions. The primary emulsions were immediately added to an aqueous solution containing carboxylic PEO-PPO-PEO block copolymer (5% w/w) which was synthesized according to the previous study (Sun et al., 2011), followed by a rapid stirring for an hour to obtain the final product of RIPNDs. The RIPNDs were washed twice with deionized (DI) water and stored in 4°C until use. The procedure of the RIPND fabrication is illustrated in Figure 1.

The morphology of the RIPND was detected using a scanning electron microscope (SEM). The surface charge and size distribution of the nanodroplets were measured by dynamic light scattering (DLS). The encapsulation efficiency (*E*_e_) of RIF or ICG was evaluated using the formula:

(1) $$E_{e}=\frac{W_{0}-W_{f}}{W_{0}}\times 100\%$$

where *W*_o_ is the amount of agent (RIF or ICG) originally used for the RIPND fabrication. *W_f_* represents the amount of free agent molecules detected in the supernatant. The separation of the RIPNDs and the supernatant was conducted by centrifugation with 6000 × *g* for 30 min. Both *W*_o_ and *W_f_* were determined by spectrophotometry (λ_abs_ = 475 and 780 nm for RIF and ICG, respectively) based on Beer-Lambert’s law. The loading rate (*R*_LD_) of the payload (RIF or ICG) in the RIPND was calculated by the formula:

(2) $$R_{\mathrm{LD}}=\frac{W_{R/I}}{W_{\mathrm{ND}}}\times 100\%$$

*W*_R/I_ denotes the weight of RIF or ICG entrapped in the nanodroplets (∼*W*_0_ × *E*_e_). *W*_ND_ is the weight of RIPND sample.

### Measurements of Stability and Release Kinetics of Entrapped Molecules

Both thermal stability of the RIPND-encapsulated ICG and release kinetics of the entrapped RIF were investigated after the RIPNDs were obtained. In this study, each RIPND sample was enclosed with a foil throughout the experiment to prevent light-induced ICG degradation (i.e., photodegradation). After incubation at 4 or 37°C for 0, 12, 24, and 48 h, the RIPNDs and the supernatant collected by centrifugation were separately measured by spectrophotometry (λ_abs_ = 780 and 475 nm for the nanodroplet and supernatant sample, respectively) to analyze the amount of ICG remained in the nanodroplets and the amount of RIF released to the bulk phase. The degradation rate coefficient (*k*_d_) of ICG in each group was calculated using a dynamic method (Saxena et al., 2004):

(3) $$\frac{C_{t}}{C_{0}}=\exp(-k_{d}\times t)$$

where *C*_0_ and *C*_t_ represent the concentrations of ICG in the RIPND at time *t* = 0 and *t* > 0, respectively. The cumulative release rate of RIF (*CR*_R_) at each time point was obtained using the formula:

(4) $${\mathrm{CR}}_{R}=\frac{A_{t}}{A_{0}}\times 100\%$$

where *A*_o_ is the amount of RIF in the RIPNDs at time *t* = 0. *A*_t_ denotes the amount of RIF found in the supernatant at time *t* > 0.

### Measurements of RIPND-Induced Hyperthermia Effect and Singlet Oxygen Generation

To evaluate the photothermal and photodynamic capabilities of the RIPND, the RIPND media with various ICG concentrations were separately exposed to an 808-nm laser with intensity of 6 W/cm^2^ immediately after placed into the well of a 96-well culture plate. The temperature of each group was measured every 30 s for 5 min using a digital thermometer, while the yield of singlet oxygen was detected using the singlet oxygen sensor green (SOSG) kit (Life Technologies, Carlsbad, CA, United States) according to the manufacturer’s instruction. The expression level of SOSG-induced fluorescence in each group was measured by spectrofluorometry every 60 s for 5 min and was quantitatively represented by relative fluorescence units (RFUs). The group of ICG solution with equal concentration settings to that in RIPND group was employed as the control.

### Microbial Cultivation

*Propionibacterium acnes* (ATCC^®^ 6919^TM^) was maintained using the reinforced clostridium medium (RCM) at 37°C under an anaerobic atmosphere (80% N_2_, 10% CO_2_, and 10% H_2_). *Staphylococcus epidermidis* [*S. epidermidis* (ATCC^®^ 12228^TM^)] was maintained using the tryptic soy broth (TSB) at 37°C under an aerobic condition. Both types of microbes were quantified by using a spectrophotometer at λ_abs_ = 600 nm and its standard curve of absorbance (optical density; OD_600_) vs. colony forming units (CFUs)/mL set prior to the experiment. Overnight cultures were diluted 1: 100 and proceeded cultivation until the OD_600_ value of the sample reached ≥ 1.0.

### Evaluation of Effect of RIPNDs on Microbial Fermentation

To examine the effect of RIPNDs on fermentation efficiencies of *S. epidermidis* and *P. acnes*, both types of microbes with 1 × 10^6^ CFUs/mL were cultured with and without RIPNDs at 37°C in the rich medium that was composed of 10 g/L yeast extract, 3 g/L TSB, 2.5 g/L K_2_HPO_4_, 1.5 g/L KH_2_PO_4_, and 0.002% (w/v) phenol red. The supernatant of each sample was subjected to spectrophotometric analysis (λ_abs_ = 562 nm) every 30 min for 3 h. The microbial fermentation efficiency (*E*_F_) was quantitatively evaluated as the variation of OD_562_ value (ΔOD_562_) detected over the time frame (Δ*t* = 3 h):

(5) $$E_{F}=\frac{\Delta {\mathrm{OD}}_{562}}{\Delta t}$$

### Examination of Effect of RIPND-Mediated Probiotic Inhibition on *P. acnes* Growth

To examine the effect of fermentation products of *S. epidermidis* induced by the RIPNDs on *P. acnes* growth, 1 × 10^9^ CFUs/mL of *S. epidermidis* were first incubated with RIPNDs containing 1.25, 2.5, 5, 10, 20, or 40 μM of ICG in a rich medium at 37°C for 12 h. The supernatant of each group that contained the microbial fermentation products was then collected by centrifugation and used for *P. acnes* cultivation afterward. The amount of survival *P. acnes* in each group was detected by (1) spectrophotometry at λ = 600 nm after 24-h incubation with the fermentation product medium (FPM) and (2) colony assay after the FPM-treated microbes were placed on the RCM agar plates for 72 h.

### *In vitro* Antimicrobial Efficacy of RIPNDs

To evaluate the antimicrobial capability of the RIPNDs, the *P. acnes* in 1 × 10^6^ CFU/mL were separately treated with ±NIR, free ICG + NIR, free RIF, RIPNDs ± NIR, and FPM ± (RIPNDs + NIR) under different procedures. NIR exposure was performed using an 808-nm laser with output intensity of 6 W/cm^2^ for 5 min. Treatment of free RIF means that the *P. acnes* were incubated with naked RIF for 24 h. Treatment of free ICG + NIR means that the *P. acnes* were exposed to NIR for 5 min in the presence of naked ICG. In this study, the concentrations of free RIF and ICG were determined based on the dosages provided by the RIPNDs and those were 0.24, 0.47, 0.95, 1.9, 3.8, and 7.6 μM for RIF and 1.25, 2.5, 5, 10, 20, and 40 μM for ICG, respectively. FPM was produced from the *S. epidermidis* (10^9^ CFUs/mL) and was collected by centrifugation after co-culture with the RIPNDs for 3 days. The six aforementioned dosages of RIPNDs were separately used for *S. epidermidis* fermentation in this study. FPM ± (RIPNDs + NIR) denotes that the *P. acnes* were solely incubated with the FPM for 12 h followed by the treatment of RIPNDs + NIR if there was. The concentration of RIPND utilized for *S. epidermidis* fermentation was equal to that used in the nanodroplet-mediated antimicrobial examination. The *P. acnes* with free RIF or sole FPM treatment were directly subjected to viability analysis immediately after the 24-h chemical stimulation, while the microbes with NIR exposure were first recovered in a 37°C anaerobic condition for an additional 24 h then subjected to viability analysis afterward. The antimicrobial susceptibility of each treatment was determined based on the value of microbial population index [i.e., Log_10_ ((CFU + 1)/mL)] that the CFU of the *P. acnes* was counted after the microbes were placed on the RCM agar plates for 72 h.

### Statistical Analysis

All data were acquired from three independent experiments and are presented as the mean ± standard deviation (s.d.). Statistical analyses were conducted using MedCalc software in which in which comparisons for one condition between two groups were performed by using one-way analyses of variance (ANOVA) with a significance level of *P* < 0.05 throughout the study.

## Results and Discussion

### Characterization of RIPNDs

**FIGURE 2:**
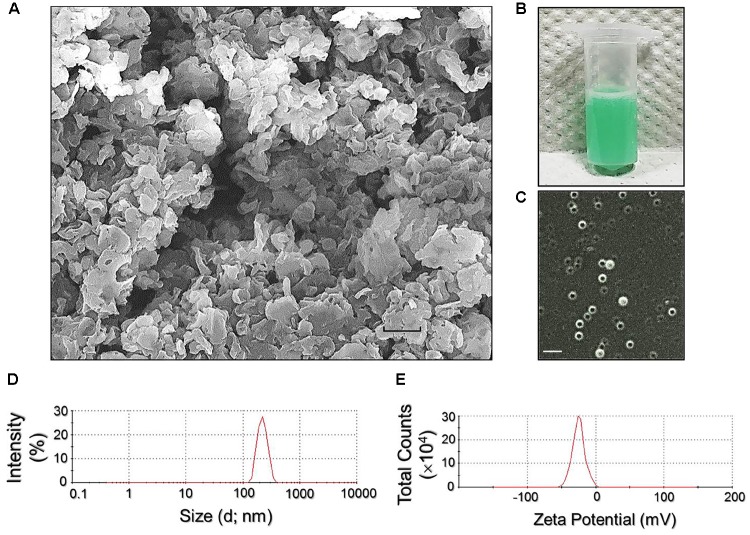
Assessment of physicochemical properties of the RIPNDs. **(A)** SEM image of the RIPNDs at 2000X magnification. Scale bar = 500 nm. **(B)** The appearance of the real RIPND sample. **(C)** Photomicrographic image of the RIPNDs at magnification of ×400. Scale bar = 3 μm. **(D)** Size distribution profile of the RIPNDs measured by DLS. **(E)** Surface charge (ζ-potential) distribution profile of the RIPNDs detected by DLS.

Figure 2A exhibits the SEM image of the RIPNDs where it can be seen that the produced nanodroplets retained intact particulate shape without collapse after the fabrication procedures including high-speed centrifugation and agitation. The green-to-orange emulsified appearance of the RIPND sample (Figure 2B) implies that the RIF and ICG were in the nanodroplets. Moreover, the double-layer structure of the RIPND can be clearly identified according to the photomicrographic image of the sample as shown in Figure 2C. Based on the DLS analyses, the size and the ζ-potential of the RIPNDs were 240.7 ± 6.73 nm (Figure 2D) and −20.9 ± 2.40 mV (Figure 2E), respectively. The negative surface charge of the RIPND was reasoned that because excessive carboxylic moieties provided by the acid-terminated PEO-PPO-PEO block copolymer were distributed on the nanodroplets’ surface. Through the calculations of Equation (1) and Equation (2), the encapsulation efficiencies of RIF and ICG in the RIPNDs were 54.0 ± 10.5% and 95.0 ± 4.84%, respectively, whereas their loading rates were approximately 0.12 ± 0.04 wt‰ and 0.64 ± 0.22 wt‰, respectively.

### Thermal Stability of RIPND-Entrapped ICG and Release Rate of RIF

**FIGURE 3:**
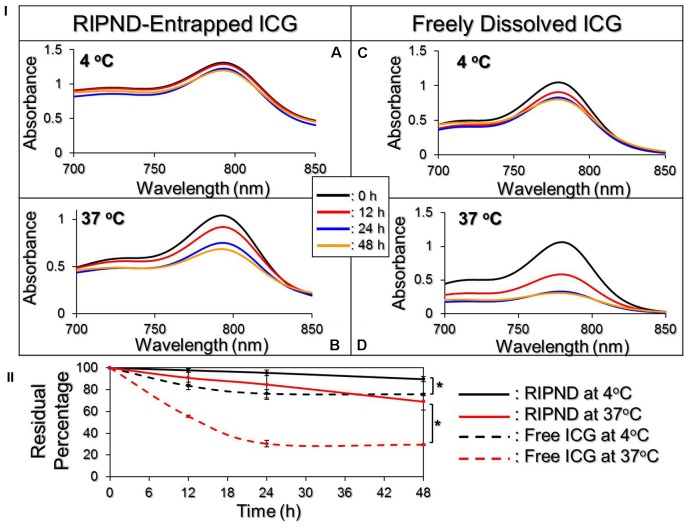
Evaluation of the thermal stability of freely dissolved ICG and RIPND-entrapped ICG. **(I)** UV-Vis spectra of freely dissolved ICG in DI water **(A,B)** and RIPND-entrapped ICG **(C,D)** under 4 **(A,C)** and 37°C **(B,D)** without light exposure for 0, 12, 24, and 48 h. The absorbance at λ = 780 nm indicates the amount of ICG remaining in each sample at the time of measurement. **(II)** Quantitative analyses of residual ICG in the matrix under 4 or 37°C within 48 h. Values are mean ± s.d. (*n* = 3). ^∗^*P* < 0.05.

Figure 3I exhibits the degradation profiles of the RIPND-entrapped ICG (Figures 3IA,B) and freely dissolved ICG (Figures 3IC,D) under incubation at 4 or 37°C in the dark for 48 h. According to the spectrophotometric analyses as plotted in Figure 3II, the results show that the residual amount of ICG in the RIPNDs at 4 and 37°C markedly enhanced 1.2- and 2.3-fold (*P* < 0.05 for each), respectively, compared with that in the DI water within 48 h. Moreover, based on the analyses of degradation rate coefficient as shown in Table 1, the anti-degradability of the RIPND-entrapped ICG was approximately 3.3-fold (*P* < 0.05), higher than the freely dissolved ICG under equal heating treatment for 48 h. These results indicate that the RIPND is certainly able to provide an improved thermal stability to the encapsulated ICG molecules.

**Table 1:** Analyses of the residual percentages and the degradation rate coefficients of RIPND-entrapped ICG and freely dissolved ICG under 4 or 37°C incubation for 48 h.

Treatment^†^	ICG degradation (%)	*K*_d_ (h^−1^)
RIPND-entrapped ICG		
4°C	10.42%^∗^	0.0060^∗^
37°C	31.07%^∗^	0.0156^∗^
Freely dissolved ICG		
4°C	24.28%	0.0190
37°C	70.54%	0.0514

*^†^Treatments were all performed in the dark. ^∗^P < 0.05 compared to the value gained from the group of freely dissolved ICG under the same temperature settings.*

**FIGURE 4:**
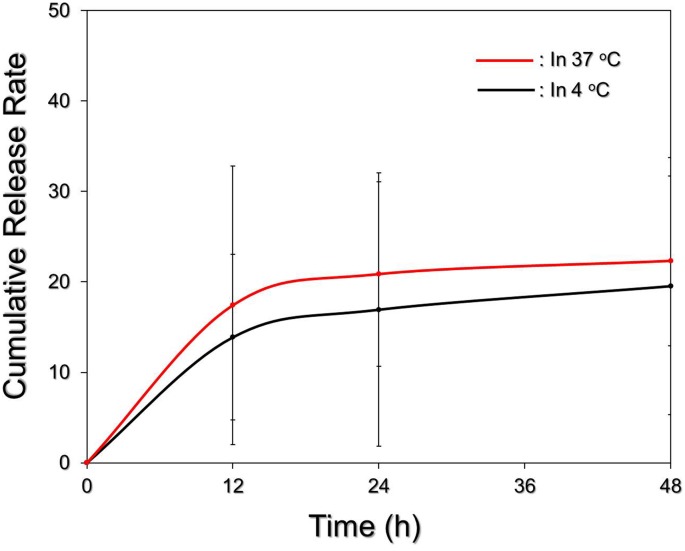
Kinetic release profiles of RIF from the RIPNDs *in vitro*. The cumulative release curves of the RIPND-entrapped RIF under 4 and 37°C incubation were established by measuring the concentrations of RIF in the supernatant at 0, 12, 24, and 48 h by spectrophotometry (λ = 475 nm). Values are mean ± s.d. (*n* = 3).

Figure 4 exhibits the cumulative release profiles of the RIPND-entrapped RIF under different temperature treatments for 48 h. Both groups expressed a biphasic drug release profile that was consistent with a number of studies (Chittasupho et al., 2009; Vivek et al., 2014), and the overall release rates after incubation at 4 and 37°C for 48 h were about 19.5 and 22.4%, respectively. Such similar drug release rates imply that the integrity of the RIPND is not sensitive to temperature fluctuation between 4 and 37°C whereby ∼80% of entrapped RIF can be successfully saved in the nanostructure within 48 h. We reason that the burst release of RIF in the first 12 h was resulted from demulsification of RIPND including phase inversion/separation, coalescence, and/or Ostwald ripening of the emulsion particles. The change of emulsion configuration may subsequently reach an equilibrium state and thus led to a decreased drug release rate afterward. In comparison with other polymeric nanostructures reported previously (Hu et al., 2012; Manca et al., 2012; Mohseni et al., 2015), the RIF is relatively stable in the RIPND and we speculate that it is attributed by (1) double layer constrain of the RIPND, (2) a less reactivity of the RIPND since its charged surface (Figure 2E) may diminish the interactions with foreign molecules and/or to each other through electrostatic repulsion that may confer an enhanced shelf stability to the nanostructure, and (3) a higher degree of steric hindrance on the nanodroplet’s surface established by tangled PEO-PPO-PEO block copolymers.

### Effects of Hyperthermia and Singlet Oxygen Generation of RIPNDs

**FIGURE 5:**
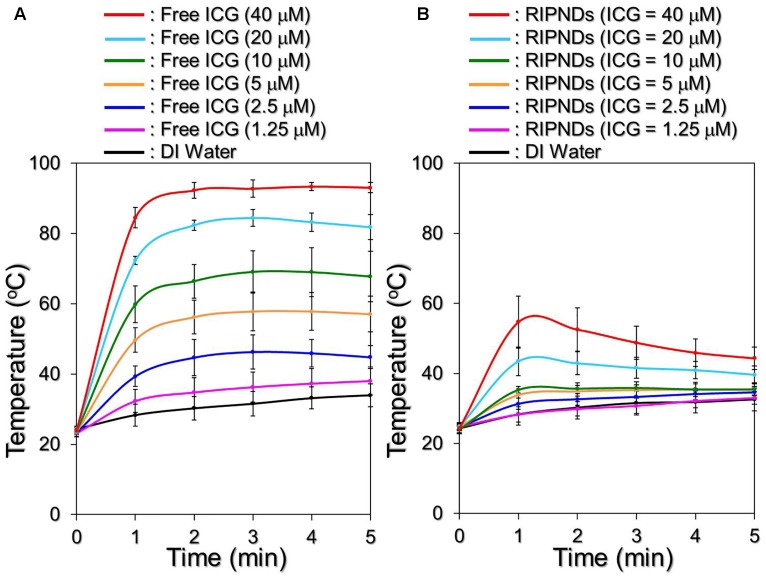
Assessments of ICG solution- and RIPND-induced hyperthermia effects under NIR exposure. Upon NIR laser irradiation (808 nm; 6 W/cm^2^), the variations of temperature in the samples of freely dissolved ICG **(A)** and RIPNDs **(B)** with equal ICG concentration settings of 0, 1.25, 2.5, 5, 10, 20, and 40 μM were separately measured using a digital thermometer every 1 min for 5 min. Values are mean ± s.d. (*n* = 3).

Figure 5 shows the hyperthermia effects of free ICG (Figure 5A) and RIPNDs (Figure 5B) with various concentrations in 5-min NIR exposure (808 nm; 6 W/cm^2^). Similar with the ICG solution, the temperature in the RIPND sample quickly elevated in the first minute and sustained in the similar level (groups with ≤10-μM ICG) or slowly declined (groups with ≥20-μM ICG) afterward, obtaining an increase of 8.1, 8.2, 10, 10.7, 11.5, 14.9, and 20°C after 5-min NIR irradiation for the RIPNDs with 0- (DI water only), 1.25-, 2.5-, 5-, 10-, 20-, and 40-μM ICG, respectively.

However, one may notice that the temperature level achieved by the RIPND group was lower than that obtained from the freely dissolved ICG under the same ICG concentration setting. We speculate that it was because different from the freely dissolved ICG where all the ICG molecules were able to simultaneously react upon NIR exposure, the hyperthermia effect of the RIPNDs can only be achieved by partially released ICG. Furthermore, demulsification occurred during NIR irradiation is a process of heat absorption (Ferreira et al., 2013) and that may affect/diminish the thermal energy given to the solvent. Therefore, the magnitude of RIPND-induced temperature elevation was relatively moderate compared with that induced by freely dissolved ICG (Figure 5B). Nonetheless, these outcomes clearly demonstrate that the RIPNDs are able to generate a dose-dependent hyperthermia effect upon NIR exposure.

**FIGURE 6:**
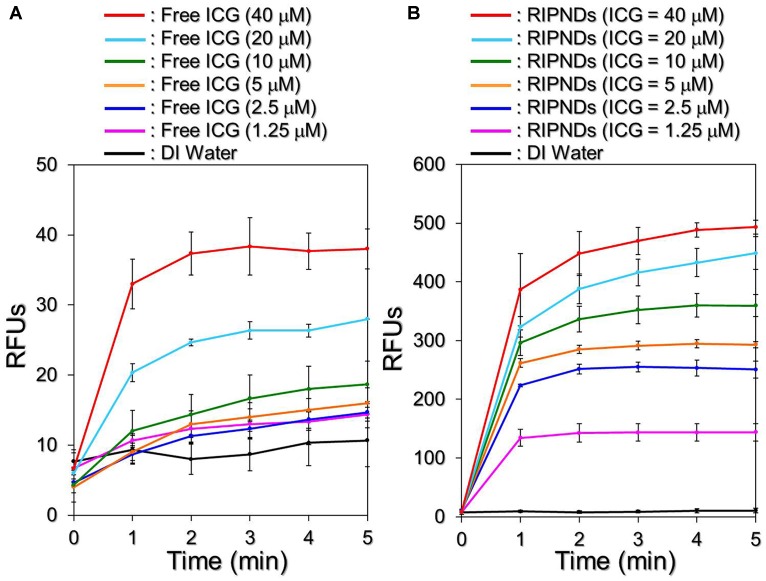
Assessments of ICG solution- and RIPND-induced singlet oxygen productions under NIR exposure. Upon NIR laser irradiation (808 nm; 6 W/cm^2^), the amount of singlet oxygen generated from the freely dissolved ICG in DI water **(A)** and RIPNDs **(B)** with equal ICG concentration settings of 0, 1.25, 2.5, 5, 10, 20, and 40 μM were separately measured every 1 min for 5 min. The quantity of singlet oxygen in each sample was analyzed based on the SOSG-induced fluorescence intensity measured by using a spectrofluorometer with 488-nm excitation wavelength and 525-nm emission wavelength, and was quantitatively represented by RFUs. Values are mean ± s.d. (*n* = 3).

Figure 6 exhibits the effects of singlet oxygen production generated from various concentrations of the freely dissolved ICG (Figure 6A) or RIPNDs (Figure 6B) within 5-min NIR exposure. Our data show that the RIPNDs certainly enabled a dose-dependent production of singlet oxygen in the dose range of 0 – 40-μM ICG as it was performed by using free ICG. However, the RIPND-induced singlet oxygen yield was exceptionally higher than that gained from the same concentration of free ICG. Based on the RFU analyses, the yields of singlet oxygen generated from the RIPNDs were 10-, 17.1-, 18.3-, 19.3-, 16-, and 13-fold (*P* < 0.05 for each) higher than those obtained from the freely dissolved ICG when the concentrations of ICG were set as 1.25-, 2.5-, 5-, 10-, 20-, and 40-μM, respectively. These results clearly show that the RIPNDs were able to provide an enhanced amount of singlet oxygen compared with equal concentration of free ICG upon NIR exposure. We reason that such improved photodynamic efficacy was attributed to the constituent PFC (PFOB) since it may allow RIPNDs to carry increased amount of oxygen that is greatly favorable for singlet oxygen production.

Due to its advantages of effectiveness, safety, and minimal complication, the use of light in the acne treatment has gained increasing attention in the last decade. So far, a variety of modalities such as narrowband light sources, intense pulsed light, and/or lasers have been widely investigated, and treatments with those lighting approaches may offer improvements in inflammatory acne and/or acne scarring, but provide limited benefit for non-inflammatory acne (comedonal) (Pei et al., 2015). Similar with the PDT, the primary means of acne eradication by PTT is acute microbial membrane destruction, wherein the temperature level plays the key role in the efficacy of thermal therapy. According to previous studies, the optimal temperature for *P. acnes* growth is between 30 and 37°C (Achermann et al., 2014); while their growth may slow down in room temperature or completely stop when the environmental temperature is ≥45°C (Szmygin et al., 2014). Although the elevated temperature may offer more opportunities to impair/inhibit *P. acnes*, a moderate temperature setting in 41 – 43°C is rather preferably used in the clinic in order to avoid any possible heating-induced damages such as water vaporization, desiccation, and/or carbonization in the surrounding cells/tissues (Coffey et al., 2006). Based on the results shown above, the RIPNDs with ≥20-μM ICG were able to offer both photothermal (T > 41°C) and photodynamic effects for *P. acnes* eradication under NIR exposure (808 nm; 6 W/cm^2^), while those with <20-μM ICG can solely provide photodynamic functionality without hyperthermia effect (T < 40°C; Figure 5B).

### Effects of RIPNDs on Microbial Fermentation Efficiency

**FIGURE 7:**
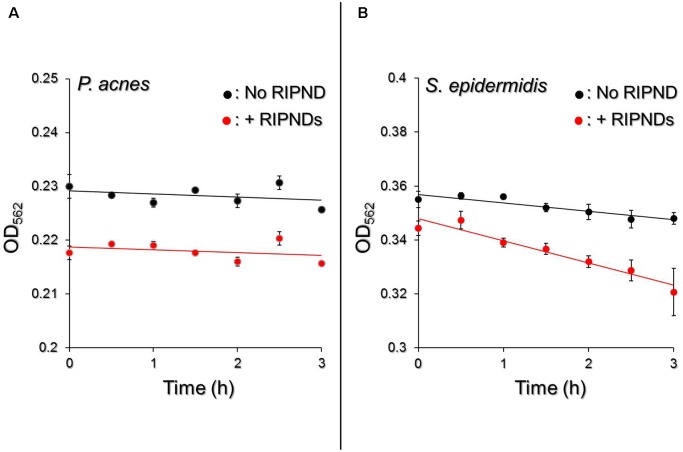
Effect of RIPNDs on microbial fermentation efficiency. Upon incubation at 37°C in the phenol red-containing rich medium, the fermentation levels of *P. acnes* **(A)** and *S. epidermidis* **(B)** in the presence and absence of RIPNDs were separately measured by using a spectrophotometer (λ_abs_ = 562 nm) every 30 min for 3 h and were quantitatively represented by OD_562_. The solid line denotes the linear trendline of the OD values obtained in each group. Values are mean ± s.d. (*n* = 3).

Although the constituent of the RIPND surface; the PEO-PPO-PEO block copolymer which is a PEG derivative, has been demonstrated as a feasible material for microbial fermentation (Frings et al., 1992; Kao et al., 2016), two issues that (1) the availability of the RIPND entity for microbial fermentation and (2) whether the RIPNDs can serve as a fermentation inducer for the probiotics but not *P. acnes*, still need to be addressed before applying the nanodroplets to bactericidal application. Based on the spectrophotometric analyses as shown in Figure 7, our data show that *P. acnes* with and without RIPNDs exhibited similar fermentation rates within 3-h incubation at 37°C (*E*_F_ = 0.00057 vs. 0.00051; *P* = NS, Figure 7A), while that of *S. epidermidis* in the presence of RIPNDs significantly enhanced 2.6 folds (*P* < 0.05) compared with the one without RIPNDs (Figure 7B). These results clearly show that the RIPNDs were able to selectively induce *S. epidermidis* fermentation, but not on *P. acnes*. Since the fermentation product of *S. epidermidis* has been known to be able to arrest the growth of *P. acnes* (Wang et al., 2014), the developed RIPND is highly potential for use in probiotics-mediated *P. acnes* inhibition.

### Antimicrobial Capability of RIPNDs to *P. acnes*

**FIGURE 8:**
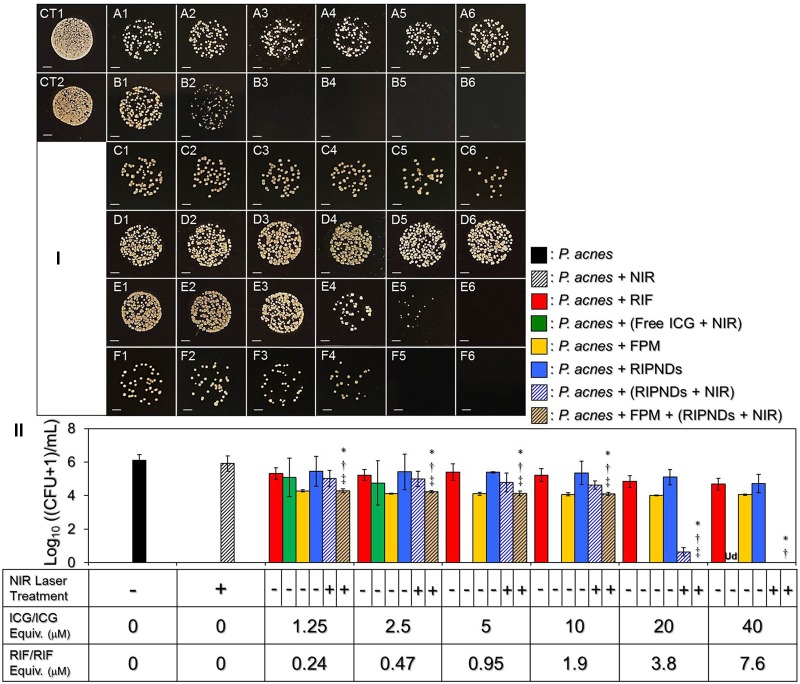
The antimicrobial efficacy of the RIPDNDs to *P. acnes*. **(I)** Photographic images of *P. acnes* colonies after treated with various conditions. Rows **(A – F)** represent the groups that cells were treated with free RIF (row **A**), free ICG + NIR (row **B**), FPM (row **C**), RIPNDs (row **D**), RIPNDs + NIR (row **E**), and FPM + RIPNDs + NIR (row **F**), respectively. The columns 1–5 denote that the bacteria were treated with free RIF in 0.24, 0.47, 0.95, 1.9, 3.8, or 7.6 μM (row **A**), respectively, free ICG in 1.25, 2.5, 5, 10, 20, or 40 μM (row **B**), respectively, FPM stimulated by RIPNDs with combined RIF/ICG in 0.24/1.25, 0.47/2.5, 0.95/5, 1.9/10, 3.8/20, or 7.6/40 μM (rows **C**), respectively, or RIPNDs with aforementioned dose settings (rows **D–F**). CT1 denotes that the bacteria were treated with neither compound (ICG and/or RIF) nor NIR exposure. CT2 represents that the bacteria were treated with NIR alone for 5 min followed by incubation at 37°C for 24 h. Each image represents the colony forming condition of each group using 1000-fold diluted *P. acnes* as the initial bacteria seed after cultivation in the agar plate for 3 days. All images were photographed using an optical microscope at 200X magnification. Scale bar = 2 mm. **(II)** Quantitative analyses of the colony number of *P. acnes* after treatment with free RIF, ICG, FPM, and/or RIPNDs in different conditions as indicated in the *X*-axis. Values are mean ± s.d. (*n* = 3). ^∗^*P* < 0.05 compared to the group with equal concentration of free RIF. ^†^*P* < 0.05 compared to the group with equal dose of RIPNDs without NIR exposure. ^‡^*P* < 0.05 compared to the group with equal dose of RIPNDs and NIR irradiation.

Figure 8I shows the colony formation of *P. acnes* after treated by ICG, RIF, FPM, and/or RIPND in different dosages with and without NIR laser irradiation (808 nm, 6 W/cm^2^). The concentrations of free ICG and RIF examined in the bactericidal experiments were corresponding to the dosages provided by the RIPNDs. Based on the CFU analyses (Figure 8II), it can be seen that the population index of the *P. acnes* treated with NIR alone [Figure 8I, CT2, Log_10_ ((CFU + 1)/mL) = 5.9 ± 0.454] was similar with that in the blank setting [Figure 8I, CT1, Log_10_ ((CFU + 1)/mL) = 6.11 ± 0.347; *P* = NS], indicating that the slight temperature increase of the medium due to NIR irradiation (Figure 5) was non-toxic. An increased bactericidal efficacy can be obtained in the free RIF- (Figure 8I, row A) or ICG- (Figure 8I, row B) treated group along with increase of drug dose, while the FPM offered a similar antimicrobial effect regardless the amount of RIPNDs used for *S. epidermidis* fermentation (Figure 8I, row C, *P* = NS). Moreover, the results show that the *P. acnes* treated with FPM followed by RIPNDs + NIR (Figure 8I, row F) underwent higher mortality compared with that treated by (1) RIF alone, (2) RIPNDs without NIR exposure, and/or (3) RIPNDs + NIR without FPM (*P* < 0.05 for all comparisons when the dose of the RIPND was ≤20-μM ICG/3.8-μM RIF), and none of colony can be obtained when the employed RIPNDs was in the dosage of ≥20-μM ICG/3.8-μM RIF as displayed in Figure 8I, F5 and F6. These outcomes clearly show that the RIPNDs are certainly effective on *P. acnes* eradication upon NIR irradiation (808 nm; 6 W/cm^2^) after FPM treatment, but are less toxic in the absence of NIR exposure.

In this study, we found that the ICG + NIR exhibited an extremely high anti-*P. acnes* effect that none of colony formation could be observed when the dosage was ≥5 μM (Figure 8I, B3 – B6). In addition, the RIPNDs in association with NIR exposure may provide higher bactericidal efficacy than the free RIF in each dose setting and the resulting microbial mortality was even higher than that caused by using four-fold increased amount of free RIF alone (Figure 8II). These outcomes clearly demonstrate the significance of phototherapy in anti-*P. acnes* treatment. Moreover, such bactericidal efficacy achieved by using RIPNDs + NIR can be further enhanced through the conduction of FPM pre-treatment. In this *in vitro* anti-*P. acnes* study, the use of FPM was to mimic the hypothetic probiotic effect of RIPNDs in practical *in vivo* dermal application since the *S. epidermidis* are intrinsically distributed on the skin surface and may be efficiently fermented due to presence of RIPNDs. Based on the microbial population index analysis (Figure 8II), we found that the FPMs generated using different amounts of RIPNDs exhibited similar bactericidal efficacy on *P. acnes*, indicating that the RIPND can solely increase the *S. epidermidis* fermentation efficiency in dose-independent manner, but not be able to enhance the antimicrobial capability for the produced FPM. To achieve a 100% bactericidal effectiveness to *P. acnes*, our data show that the microbes with 12-h pre-treatment of FPM can be completely eradicated using RIPNDs with 20-μM ICG/3.8-μM RIF (Figure 8I, F5), while a double amount of the RIPND was required if the FPM treatment was excluded (Figure 8I, E6). These outcomes indicate that the probiotics indeed played a crucial role in the RIPND-mediated anti-*P. acnes* treatment.

However, the RIF chemotherapeutics of the RIPNDs is indispensable because it may take over the therapeutic roles from FPM and ICG after NIR exposure and provide a relative long-term antimicrobial effect thereafter. To further enhance the anti-*P. acnes* efficacy of the developed nanodroplets, the use of different carrier materials and/or a cocktail of different chemo-drugs and/or photosensitizers in the payload may be a feasible strategy, but those approaches will certainly need to be verified through experiments. Taken all together, with merits of improved ICG stability, selective fermentability to *S. epidermidis*, and robust effect of *P. acnes* eradication, the RIPND is considered to be a novel multi-therapeutic agent for anti-*P. acnes* application and is anticipated to be able to provide less antibiotics-induced chemotoxicity that is highly advantageous for use in the clinic.

## Conclusion

In this study, we have presented a proof-of-concept study of a combined photo-chemo-probiotic therapeutics through use of RIPNDs for anti-*P. acnes* treatment. We not only investigated the nanodroplet’s physicochemical properties and functionalities, but also evaluated their practical effectiveness on antimicrobial application *in vitro*. Based on the microbial population index analyses, *P. acnes* with 1 × 10^6^ cells/mL can be completely eradicated by operation with 12-h FPM co-culturing (v/v = 1:1; probiotic therapy) followed by the treatment of RIPNDs (≥20-μM ICG/3.8-μM RIF) + NIR (808 nm, 6 W/cm^2^) for 5 min (photochemotherapy). We anticipate that such antimicrobial effect conducted by placing the RIPNDs with appropriate ICG and RIF doses on the skin surface for 12 h followed by NIR irradiation can be reproduced in human dermal (*in vivo*) study. However, we do understand that further studies are certainly required to fully address the applicability of the RIPND in the clinic. Overall, given the high prevalence of acne vulgaris in adolescents worldwide, the RIPND may provide a feasible alternative for such dermatosis. Currently we are actively conducting orthotopic murine models to examine the effect of RIPNDs on anti-*P. acnes in vivo* and aim to translate our efforts into a viable clinical strategy in the future.

## Author Contributions

K-HH performed the experiments and analyzed the data. C-MH provided advice for the study design and edited the paper. Y-HL conceived the study, provided technical advice for all experiments, and wrote the paper.

## Conflict of Interest Statement

The authors declare that the research was conducted in the absence of any commercial or financial relationships that could be construed as a potential conflict of interest.
